# Developing an Evidence-Based Patient Education Guide on Pain Management for Asian Patients on Hospice

**DOI:** 10.1097/NHH.0000000000001275

**Published:** 2024-07-08

**Authors:** Sally Huang, Joan Gygax Spicer

**Affiliations:** **Sally Huang, MSN, RN**, is a Clinical Manager, Asian Network Hospice, Oakland, California.; **Joan Gygax Spicer, MBA, PhD, RN, NEA-BC, CCM**, is an Adjunct Faculty, California State University East Bay, Hayward, California.

## Abstract

Asian Americans are the fastest-growing racial group in the United States. This article describes the development of a pain management assessment guide for Asian patients on hospice, their families, and their nurses. Thematic analysis was used to evaluate the applicability of research on pain, pain management, and barriers to pain management from primarily Asian countries to Asian patients on hospice in the United States. Thematic analysis of interviews with such patients concurs with research findings. Four themes emerged: enduring pain, preference for Chinese medicine remedies, fear of addiction, and concern about the side effects of pain medications. Interviews with experienced hospice nurses also aligned with these themes. Hospice nurses were asked to share their strategies for assessing and managing pain among their Asian hospice patients. Thematic analysis of their interviews revealed six strategies: focusing on treatment goals, involving family and caregivers, explaining the physiology of pain, explaining the progression of pain medications, addressing concerns about addiction, and managing the side effects of medications. The themes that emerged from patient and hospice nurse interviews were used to develop an evidence-based pain management assessment guide to support Asian patients on hospice, their family, and the nurses who care for them.

**Figure:**
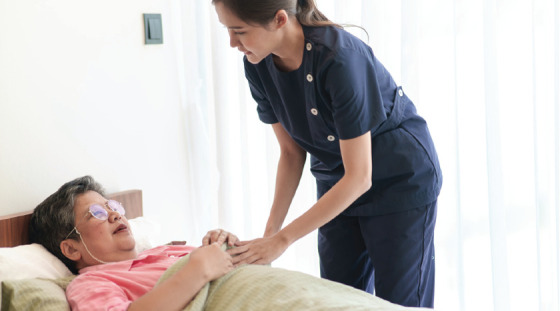
No caption available.

Between 2000 and 2015, the population of Asian Americans in the United States increased by 72%, making it the fastest-growing racial group in the country (Lee et al., 2021). Fifty-nine percent of Asian Americans were born in another country and since 1965, one fourth of all immigrants to the United States have come from Asia (Lee et al., 2021). Just over 12% of the U.S. population is Asian (United States Census Bureau, 2023). Living and working in an ethnically and culturally diverse society requires that nurses develop evidence-based, culturally sensitive interventions. This project began with a review of research on pain, pain management, and barriers to pain management among Asian patient populations. The applicability of the findings to the care of U.S. Asian patients on hospice was evaluated using thematic analysis of interviews with patients and their nurses. The outcome of this project was the development of an evidence-based, pain management assessment guide for Asian hospice patients.

The setting for this project was a home healthcare and hospice agency, which was staffed by ethnically diverse nurses who serve a primarily Asian population. A significant “brain trust” of knowledge and cultural competency exists within this staff. Unknown, however, was whether the nurses' experience reflects evidence-based practice and whether one nurse's experience represents the collective experience of other nurses in guiding pain management for Asian patients. We first turned to the literature for evidence-based nursing practices on pain management for primarily Asian patients but found little guidance. Our focus then shifted to exploring the applicability of international research on pain among U.S. Asian patients on hospice. This review evolved into developing an evidence-based pain management assessment guide for this patient population, which was informed by our Asian patients and diverse nursing staff.

A review of the literature included English-language articles on studies in Asian countries (China, Japan, Korea, and Taiwan) of terminally ill adult patients, advanced diseases, pain management, barriers to pain medication adherence, and the perceptions of healthcare providers on patient noncompliance with pain control regimens. Because we focused on published research with Asian samples versus subgroups of Asian populations within samples, findings were limited, even after the search was extended past 10 years.

Five quantitative studies and one qualitative study were selected for in-depth review. The review focused on patient-centered variables, the relationships between these variables, and measurement tools. Disease-treatment variables and system or medical provider variables were excluded from our review (Table 1).

**Table 1.** Research on Pain Management Variables among Asian Patients

Authors	Country	Patient Pain Management Variables	Measurement Tools
Liang et al. (2013)	Taiwan	Higher scores on opioid. Analgesic beliefs have a negative relationship with adherence to the opioid analgesic regime.	Brief Pain Inventory-Chinese Pain Opioid Analgesics Beliefs Scale-Cancer
Duke and Petersen (2015)	United States	**Japanese** -Pain is part of dying. -Hesitancy to express pain. -Pain medication is not acceptable. **Chinese** -Fear of burdening the provider. -Fear side effects and addiction. -Pain is a personal matter. -Do not want to burden others. -Endure the pain until excruciating. -Lack of knowledge. **Vietnamese** -Did not want to be a burden. -Wanted to be awake and alert. -Endure pain cultural and spiritual. -Trust the healthcare provider. -Alternatives to medication.	Thematic qualitative.
Shen et al. (2017)	Taiwan	Negative Scores on Pain Management Index related to lower scores on the quality-of-life domains: -General activity. -Normal work. -Walking. -Sleep. -Enjoyment of life. -Relations with other persons.	Pain Management Index **Brief Pain Inventory**
Xia (2017)	China	Patient barriers to pain management related to: -Fear of side effects. -Fear of addiction. -Reluctance to report pain. Pain affects daily life activities: -Sleeping. -Concentration and focus. -Forced to rely on other people. -Activities of daily living. -Not allow a good quality of life.	ACHEON Survey Pain Severity Box Scale
Zeng et al. (2020)	China	Medication adherence and analgesic use relate to: -Concerns for tolerance. -Concerns about side effects. -Good patient does not complain. -Distract doctor from treatment. -Concerns about addiction. -Fatalism cancer pain cannot be controlled. College educational level related to: -Increased concern about side effects. -Decreased concern about good patient. Female gender related to: -More concern about tolerance. -More concern about good patient. -More concern about distracting physician.	Barriers Questionnaire-Chinese Morisky Medication Adherence Scale Pain Management Index
Ma et al. (2020)	China	Attitudinal barriers related to pain management: -Concerns about tolerance. -Concerns about addiction. -Pain endurance.	Barriers Questionnaire-Chinese Self-Reported Adherence to Opioid Prescriptions

The Barriers Questionnaire-Chinese, an instrument that examines patient-related barriers to managing pain provided our study with an organizing framework to evaluate the applicability of international research to local Asian patients on hospice. The questionnaire measures seven domains: (a) fear of addiction to analgesics, (b) concern about the side effects of analgesics, (c) concern about tolerance to analgesics, (d) fatalism that pain cannot be controlled, (d) concern that increased pain means disease progression, (f) a desire to be a good patient by not complaining about pain, and (g) concern about distracting physicians from the cancer treatment (Zeng et al., 2020). Two studies in our review used the Barriers Questionnaire-Chinese (Ma et al., 2020; Zeng et al., 2020). Both found medication adherence was related to concerns about tolerance, addiction, and enduring pain. In addition, Zeng et al. (2020) found medication adherence and analgesic use were related to (a) concerns about side effects, (b) a good patient does not complain, and (c) distracting physicians from treatment. Xia (2017) identified patient barriers to pain management as fear of side effects, fear of addiction, and patient reluctance to report pain.

Using the Pain Opioid Analgesics Beliefs Scale-Cancer, Liang et al. (2013) found opioids had a negative relationship with adherence to an opioid regime. The instrument, a 10-item questionnaire, uses a Likert-type scale that ranges from 1 (*strongly agree*) to 5 (*strongly disagree*). Beliefs about analgesics relate to adherence to an analgesic regime. Questions about analgesic beliefs addressed these concerns: opioid medicine is not good for one's body; opioid medicine should only be used at the last stage of an illness; initiation of opioid medicine means health is already in serious condition; opioid medicine causes many side effects; side effects are not easy to handle; adults should not ask for pain medicine frequently; the more one uses opioid medicine, the greater the possibility that they might rely on it forever; initiation of opioid medicine at too early a stage will have less of an effect later on; and adults should endure as much pain as possible. Shen et al. (2017) and Xia (2017) identified the extent to which pain interferes with quality of life. Xia (2017) included the domains of sleeping, concentration, and activities of daily living. Using the Brief Pain Inventory, Shen et al. (2017) studied the domains of general activity, normal work, walking, sleep, enjoyment of life, and relations with other people.

Some researchers have used other instruments to measure pain and adherence to prescribed medication regimes. For pain, Shen et al. (2017) and Zeng et al. (2020) used the Pain Management Index; Xia (2017) used the Box Score-11 Scale. For adherence to medication regimes, Ma et al. (2020) and Zeng et al. used the Morisky Medication Adherence Scale.

Using a descriptive qualitative approach, Duke and Petersen (2015) interviewed Japanese, Chinese, and Vietnamese people, who were living in Texas, to elicit their views on pain management in the final days of life. Thematic analysis revealed three overarching themes for the Japanese group and five themes each for the Chinese and Vietnamese groups. The Japanese group revealed:

1. Pain was natural and a part of dying, and people were not supposed to accept pain medications.
2. They were hesitant to express pain.
3. Pain medication was not acceptable because it was believed to be bad and unhealthy.

(Duke & Petersen, 2015, p. 28-29)

The Chinese group revealed:

1. Pain was often not communicated by patients or family out of fear of burdening the healthcare provider.
2. Pain was a private matter. Patients might be more accepting of pain medications if family members who opposed it were unaware of their situation.
3. The fear of side effects, especially the potential loss of speech and fear of addiction, deterred patients from taking pain medications.
4. Patients endured pain and waited until it was severe or excruciating to request pain medications.
5. Unaware the administration of opioids could be safe and effective, patients refused pain medication unless they knew the cause of pain and how medication would help.

(Duke & Petersen, 2015, p. 29-30)

In the Vietnamese group, the researchers found:

1. Patients did not volunteer they had pain because they did not want to be a burden.
2. Patients wanted to be alert in the final days of life to be able to communicate with their family.
3. Patients felt the need to endure pain because it is a cultural value and spiritual expectation.
4. Medication adherence depended on trust that the healthcare provider prescribed the correct pain medication.
5. Patients preferred alternative therapies rather than medication for pain management.

(Duke & Petersen, 2015, p. 30-3129)

## Methods

This project had three aims: (a) to evaluate the applicability of Asian research to Asian patients on hospice in the United States; (b) to identify pain management and assessment strategies hospice nurses can use to support such patients; and (c) to develop an evidence-based pain management assessment guide for Asian patients on hospice, based on research, our Asian hospice patients, and experienced home care and hospice nurses who work with Asian patients.

### Design

This qualitative study relied on field notes from patient interviews and thematic analysis of those conversations (Kiger & Varpio, 2020). Recording the interviews during home visits was considered. However, in the light that China was suspected of being the origin of COVID-19 and that the resultant societal and governmental climate in the United States made them uncomfortable, recording was dropped. Moreover, Rutakumwa et al. (2020) found the quality of data from voice-recorded transcripts and from field notes written directly after interviews was comparable. This project was reviewed and approved by the California State University East Bay Institution Review Board. Informed consent was obtained from patients and hospice nurses for voluntary participation in the interviews.

### Framework

Based on the literature review, a set of questions was created to explore and evaluate the applicability of international research to a community-based Asian population on hospice. In addition, nurses were asked to share their insights and strategies for pain assessment and management of Asian patients on hospice.

### Setting and Recruitment

This study was conducted at a community-based hospice that serves Asian patients in Oakland, CA. The county's Asian population is 23.4% of the total population. Criteria for inclusion included: documented pain, prescribed pain medication, and a terminal diagnosis. Eligible patients were called and if oral consent was received, interviews were scheduled, and written consent was obtained. Patients were called sequentially until the interview field notes reached saturation in recurring themes. If patients had a terminal diagnosis or a diagnosis that could cause them to be cognitively impaired (e.g., brain cancer), their durable power of attorney was asked to provide consent and participate in the interview.

Hospice nurses were invited by email from the hospice agency administrator to participate in this project. Six nurses volunteered to do so. The nurse interviewer scheduled the volunteers for interviews.

## Data Collection

Patient interviews took place in their homes. The interviews, which took 20 to 60 minutes, comprised questions on their a.) pain level and how pain affected their daily activities, b.) current pain medication, and c.) understanding of opioids and narcotics. After six patient interviews, themes were reoccurring, and the sample was closed. See Table 2 for patient demographics. The six hospice nurses who volunteered to participate were interviewed by telephone. Nurse interviews were 20 to 30 minutes long using a set of questions about their insights and strategies for pain management of Asian patients on hospice. Field notes were used to collect data from the patients and nurse interviews. After field notes were finalized, they were typed up immediately. See Table 3 for hospice nurse demographics.

**Table 2.** Patient Demographics (N = 6)

Gender	
Female	4
Male	2
Age	
60-80	3
81-99	2
>100	1
Ethnicity	
Chinese	4
Japanese	1
Korean	1
Terminal Diagnosis	
Cancer	4
Noncancer diagnosis	2
Self-reported Pain Scale: 0 (no pain) to 10 (worse pain imaginable)	
6	3
5	1
Occasional pain	1

**Table 3.** Hospice Nurse Demographics

Demographic	Nurses
Gender	
Female	3
Male	3
Years of Hospice Experience	
0-2	2
3-5	2
6-10	1
>10	1
Ethnicity of Nurses	
Chinese	4
Vietnamese	2

### Thematic Analysis

Four themes related to pain and pain management were identified by the six patients and six nurses: a.) enduring pain, b.) preference for Chinese medicine remedies, c.) fear of addiction, and d.) concerns about side effects. Nurse interviews focused on their insights and strategies for managing patient pain. Six themes emerged from their interviews: a.) treatment goals of hospice, b.) involvement of family and caregivers in education, c.) explanation of the physiology of pain, d.) early education on the progression of pain medication, e.) addressing concerns about addiction, and f.) managing side effects.

### Patient Insights

Thematic analysis of patient interviews revealed four recurring themes: a.) enduring pain, b.) preference for Chinese medicine remedies, c.) fear of addiction, and d.) concern about the side effects of pain medications.

### Enduring Pain

Two patients reported only occasional pain, “Now tolerable and does not affect my life.” and “I will be in pain at the end, I can do most things still by myself” [P-5]. During interviews, two patients described how pain limited their movement. One patient reported pain at Level 6, “Can't move when I am having pain” [P-3]. Another patient remarked, “Mobility is poor due to pain” [P-2]. Another patient reflected that if pain were better, he would “walk more, get everyday life back like 10 years ago [Level 6]” [P-2]. The patient referred to his pain as being a “huge inconvenience for family” [P-2]. “Family hears the pain but cannot do anything about it” [P-2]. Another family member shared, “Family worried about how to manage pain” [Level 6]. Only one patient [Level 5] indicated pain does “not affect my life” [P-1].

Nurse observations on the impact of pain included such patient comments as, “Despite pain every day when walking, [he] still refused to take pain meds including Tylenol” [N-1]. Another observation was “A lot of patients prefer to tolerate the pain without taking any pain medications unless pain is very severe” [N-1]. Patients say they “do not like meds if they can tolerate the pain” [N-6]. Although a patient told the nurse, “Pain is tolerable,” the nurse could see the patient was “still struggling to tolerate the pain...” [N-1]. The older generation “Think that pain is part of the dying process and should suffer before dying” [N-6].

### Preference for Chinese Medicine Remedies

The most frequently mentioned medications, taken by three of the six patients, were Chinese medicine remedies: herbals [P-1], pain patches [P-2], Tiger Balm [P-1], oils [P-2] [P-4], topicals [P-4], and ginger and salt foot soaks [N-4]. Patients prefer topical pain remedies. Three patients used over-the-counter medications, Tylenol^®^ [P-2] [P-3] [P-4] and Salonpas^®^ patches [P-4]. Other medications included lidocaine patches [N-4], fentanyl patches [P-1], and morphine [P-4]. Non-medicine remedies included massage, stretching, pushing on pressure points, heat packs, and aromatherapy [P-4]. As one patient explained, “Western meds address or fix the issue, relieve pain, works quick, but only treats the symptoms, whereas traditional medicine is more holistic and treats the root of the problem, the cause” [P-4].

Nurses observed patients prefer Chinese medicine remedies, believing these remedies are a “more natural way of healing [the] body” and “Western medicine is considered more a chemical and has side effects” [N-1]. The Chinese remedies may be “mild-to-moderate [in] effectiveness” [N-1]. Patients preferred to tolerate pain unless pain was severe, “At that time, they take meds, but it's less controlled because the pain is so severe” [N-3]. Three nurses observed that Chinese remedies were not effective as cancer pain increased [N-4] [N-1] [N-3].

### Fear of Addiction

The interviewer asked patients if they had heard about opioid narcotics (e.g., morphine, Norco^®^, Vicodin^®^, or oxycodone) prescribed for severe pain. One patient's reaction to morphine was, “Morphine is a drug like marijuana...side effects, body weak, addiction” [P-1]. A second patient said they were concerned about the side effects because, “Body is weak, addiction” [P-2]. “Narcotics sound like drugs that are highly addictive. Become reliant on them” [P-4]. “In the past, opium was addictive, narcotics is in the same category, so addictive” [P-4]. Another patient stated, “Don't like medication in general, but when taking narcotics, you will get addicted to it” [P-3]. The experiences of others supported concerns about addiction, “I have a friend who is actually going through addiction and cannot live without the medication, so I don't want to take everything the doctor prescribes” [P-3]. Another patient had a family member who was injured and became addicted to morphine while being treated for the injury [P-1]. One patient said they would take not morphine because, “My uncle took morphine for one day and passed away the next day” [N-4].

Two patients were receptive to stronger medications such as opioids or narcotics. One felt their pain was not severe enough to take narcotics [P-3] because, “Narcotics are only used if pain is very severe.” Another patient stated, “I would take narcotics if I was in severe pain” [P-5].

One nurse commented, “From my experience, most patients are open to trying narcotics if they were constantly suffering, I did not have any patients that would actively refuse” [N-2]. Another nurse stated “Some [patients] feel happy taking narcotics such as morphine [to be] free from pain. [N-4] [N-6] One nurse observed that families are against narcotics because of side effects like drowsiness, sleepiness, and “thought the patient will die sooner” [N-5]. Four nurses added that patients and families are also concerned that pain medications will hasten death [N-2] [N-2] [N-5] [N-4].

Patient reaction to morphine could be strong, as one nurse noted. “Sometimes just the name morphine is very scary. Most well-known of the opioids. Don't want to take morphine, but will use oxycodone...” [N-2]

### Concerns About Side Effects

Two patients experienced side effects associated with narcotics, which included dizziness [P-1], and specific to morphine: constipation, vomiting, and oversedation [P-4]. Patients were concerned about side effects such as dizziness [P-2], vomiting, sedation, weakness [P-1] [P-2], malnutrition [P-2], and constipation [P-4]. Vomiting and oversedation were associated with morphine [P-4]. Also, patients were concerned that long-term use “will affect the liver and kidneys” [P-4] and “be harmful to the body” [P-2].

One nurse remarked, “Families and patients were concerned about the use of narcotics because of the side effects of drowsiness and sleepiness.” Another patient “Actively tried to reduce the amount they take because of drowsiness because they want to interact with others, want to be alert enough. They value the time they can spend with family than with full pain control” [N-2]. “If a patient had experienced a side effect like nausea and vomiting in the past, then they don't want to try it again” [N-3]. Of note, patients had not actually experienced many of the side effects they feared.

## Nurse Insights, Strategies for Assessment, and Patient Education on Pain Management

Nurse interviews focused on their insights and care strategies for their Asian patients on hospice. Themes included discussing treatment goals, involving family and caregivers in education, explaining the physiology of pain, educating early on the progression of pain medications, addressing concerns about addiction, and managing side effects.

### Focusing on Treatment Goals

A frequent strategy in pain management was to refer hospice patients and families to the goals of treatment [N-5] [N-6]. Explaining “...most important in hospice focus is on comfort care, tolerating pain is not good” [N-3]. “Explain to patients and families regarding the quality of life and no suffering, benefits versus disadvantages. If suffer, life has bad quality” [N-5].

### Involving Families and Caregivers

For many patients, their family makes the decisions. Educating and reeducating families is extremely important [N-1] [N-2] [N-3]. “If families themselves are receptive, they play a very important role in persuading patients to take their pain meds” [N-1]. “If families also have false beliefs, it can also be very hard to convince the patient.” “Most of hospice patients are unable to take pain meds by themselves; families and caregivers take a major role to give patients meds” [N-3].

### Explaining the Physiology of Pain

Patients “don't understand the dangers of not taking pain medications” [N-6]. “Explain physiology of pain, why not taking pain meds is not good for them” [N-6]. “If pain is not well controlled, it will be very stressful for them, increase heart rate and blood pressure, muscles become more tense and burdens their work on the heart. Affects sleep, appetite, emotion...” [N-3].

### Educating Early on the Progression of Pain Medications

Introducing the progression of pain medications early on is important. It is important to “explore all the other options before jumping to narcotics” [N-2]. “Easing them from other pain medications like Tylenol^®^ then progressing to other narcotics such as oxy or Norco^®^ before morphine” [N-4]. “If they understand the purpose of meds and importance of pain meds, let them try to see the effectiveness of the pain management, they will be more compliant to take pain meds” [N-3].

### Addressing Concerns about Addiction

Four of the six nurses discussed patient and family concerns about addiction to pain medications. “Going to opioids for the first time is a difficult step, especially for those not [having] a cancer diagnosis, fear of addiction” [N-3]. The patient being “afraid of addiction even though they are already educated” [N-4] is a barrier to pain management.

Patient trust is essential. If patients have a long-term relationship with their nurse, trust is likely to follow [N-6]. “Sometimes it helps to hear it from a doctor. Asian patients place a lot of trust in their doctors, more than the nurses. Needs someone they have respect for instead of going through preconceived notions” [N-2]. It may also help to “Have doctor talk to family.”

### Managing Side Effects of Medications

Patients “need reassurance that certain pain meds don't cause a lot of side effects. The benefits are greater than the potential side effects. Lay out clearly what potential side effects they may have, and they can stop the med any time they want” [N-1]. Inform patients and families if they try to take the medication and “if they have side effects, can educate to adjust pain medications” [N-5]. “I will give one dose during the visit and monitor for symptoms of side effects and if nothing terrible happens, and medication is effective, then they will feel more comfortable to take the med” [N-3]. “If I administer the first dose, they become more receptive. If they see how they respond to it, then they would be more open” [N-2].

### Rigor and Reflexivity

Field notes were reviewed in their entirety before data were coded. The first hospice nurse reviewer organized the field notes into similar groupings. The size of the dataset lent itself to using a table to organize the data. An advanced practice hospice nurse then reviewed the table. The final review was completed by a nurse experienced in qualitative methods who was not a hospice nurse. The challenge then was editing the labels to best convey the richness of the data. Although the patients and nurses did not use either label in their interviews, the final decision was to label the groups as “enduring pain” and “fear of addiction” because the lead hospice nurse who was Asian and who conducted the interviews and worked with this patient population felt it was more representative of what she heard in the interviews and in her work.

## Findings

In our sample, patients with pain reported moderate levels of pain. On a Pain Scale that ranged from 0 to 10, they reported their pain ranged from occasional to Levels 5 or 6. Shen et al. (2017) found a relationship between scores on the Pain Management Index indicating low pain control and quality-of-life domains. Our interviews indicated that walking or mobility and relationships with family were impacted when patients had pain. Nurse observations supported the view that patients tolerate their pain. Describing patients as tolerating pain may be similar to the finding of Ma et al. (2020) that the attitudinal barrier to pain management relates to beliefs about pain endurance. Duke and Petersen (2015) found there to be a stoicism and spiritual meaning in the Japanese culture. Patients reported their preference and use of Chinese medicine remedies. In our review of the research (Liang et al., 2013; Ma et al., 2020; Shen et al., 2017; Xia, 2017; Zeng et al., 2020). Chinese medicine remedies were not addressed. Duke and Petersen (2015) found Chinese and Vietnamese patients preferred alternative therapies to medications. When the use of narcotics for severe pain was introduced during the interviews, all patients expressed concerns about addiction. However, two patients who had concerns about addiction said they would consider taking narcotics for severe pain, consistent with Duke and Petersen's (2015) findings. These findings are also consistent with the findings among Chinese patients (Liang et al., 2013; Ma et al., 2020; Shen et al., 2017; Xia, 2017; Zeng et al., 2020). Although the Centers for Disease Control and Prevention (2021) refers to opioids as the preferred term for pain management medications, patients did not differentiate between opioids and narcotics; they associate addiction with medications. In this clinical setting, nurses use descriptions of narcotics and opioids rather than the terms themselves because patients associate both terms with addiction or illegal drugs.

Experiencing or anticipating side effects was related to patient decisions about pain management, which is consistent with the findings of Zeng et al. (2020) and Xia (2017). The findings of our patient interviews indicate the research reviewed for this project is applicable and transferable to the care of our Asian patients on hospice.

The project team reviewed the nurses' insights and strategies for assessing and educating patients about pain management. The consensus was to use that information to develop a pain management assessment guide for patients and families. The guide would combine the collective nursing experiences to provide the best pain management assessment guide for Asian patients (see Appendix “Pain Management Assessment Guide for Asian Patients on Hospice”).

**Appendix 1:**
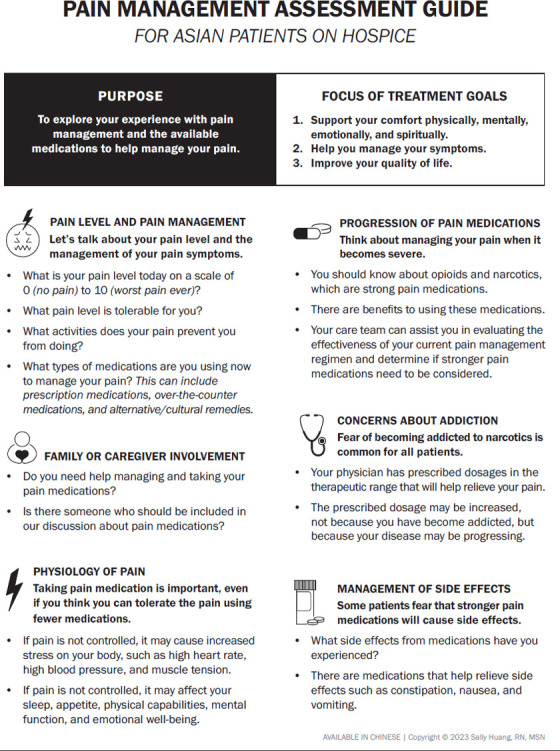
Pain management assessment guide

Nurses emphasized the need to focus on the treatment goals of hospice when discussing the effectiveness of pain management. Hay et al. (2019) found families are conflicted between the goals of hospice care to provide comfort care and their concerns about pain management and medication side effects. Most important, nurses must determine if it is the patient or the family who is making medical decisions. Healthcare providers must avoid cultural stereotypes and not assume patients are family-oriented because family is a cultural value (Alden et al., 2018).

### Strengths and Limitations

In the past 10 years, little has been published on the care of Asian patients on hospice. Our sample was recruited from one clinical site in Oakland, a large cosmopolitan city in California. Although participating patients and nurses represented several Asian groups, the generalizability of our findings and the pain management assessment guide will have to be explored with each Asian group. The Asian nurse interviewer, an experienced, bilingual hospice nurse from the community the hospice serves, felt patients may have been guarded in their responses because their comments were brief for the most part.

### Opportunities for Future Research

In a systemic review of home healthcare for Asian Americans, small sample sizes and single-site studies limited the quality evaluations of the studies (Ma et al., 2023). Future research into community-based home health and hospice services within the Asian patient population with large samples and multi-site studies would support a more comprehensive understanding of the population's needs. However, Yeung and Dong (2021) caution about aggregating diverse Asian populations in large research samples. Although securing funding for large samples and multi-site studies is beyond the purview of nurses who provide direct patient care, their efforts to investigate pain management among Asian patients in hospice, even on a small scale, should be encouraged.

## Conclusion

Healthcare providers must provide ongoing family and patient education to overcome barriers to pain management among Asian patients on hospice. The goals of hospice treatment—providing comfort and supporting quality of life—should always be in focus when nurses support patients and families in making decisions about pain management. The benefits and risks of medications for pain management and their side effects must be included in the educational process. This should impress upon patients and families that pain management and hospice treatment goals are interdependent in achieving comfort and quality of life at the end of life.
